# Address of President of the Ohio State Dental Society

**Published:** 1893-01

**Authors:** J. R. Callahan


### THE DENTAL REGISTER.

VOL. XLVII.] JANUARY, 1893. [No. 1.

### COMMUNICATIONS.

### Address of the President of the Ohio State Dental Society.

BY J. R. CALLAHAN, D.D S.

Read before the Ohio State Dental Society, December 6, 1892.

*Gentlemen of the Ohio State Dental Society*: — We have laid aside our instruments, closed our medical cases, appointment-books and ledgers, and have come to this, the twenty-sixth annual meeting of our society, to add to our stock some of the elements that go to make cultivated and successful professional gentlemen, to exchange salutations with professional brethren that we have met year after year, and whom we have learned to love as brethren.

The Ohio State Dental Society was organized in 1866 and continued under that organization to December 1884, when—for reasons known to most of you—it was reorganized and chartered, under the laws of our State. The reorganization was complete, the Constitution and By-Laws were remodeled from beginning to ending. We have now been working under this reorganization for eight years, and we can now see the wisdom of the present methods of conducting our meetings. There are weak spots in our Constitution and By-Laws that I hope will be remedied at this meeting. I desire to call your attention to the Board of Directors. This Board is of far more importance than any office or Board in the Society. The importance of electing your best men to this Board can not be overestimated. This Board is your court; it transacts all of your business. The success of our Society depends largely upon it. In the past few years they have transacted business for the Society that under the old rule would have plunged us into endless discussion and unpleasant wrangles that would have ended in profitless quarrels and enmities. The business has been done quietly and in a business-like manner. Any member can go before the Directors with any rational proposition he may desire to present, and I assure you—from an official experience of eight years that you will be received and treated with the utmost courtesy. I am of the opinion that no matter of business whatsoever should be permitted to come before the Society, except on appeal. We should ever have in mind that the best interests of the Society demand that as little time as possible should be taken from the discussion of the regular subjects; and, during this meeting, I shall, with your permission, refer all matters of a business character to the Board of Directors.

For years this Society has been trying to get a new Dental Law enacted to take the place of the old law, that has so long been absolutely useless. Committees have been appointed year after year, and as often as appointed have visited Columbus to confer with the standing committee from the legislature on Medical Colleges and Societies. The bills were always lost in the Committees. I have no recollection of any of our numerous amendments or new bills, getting far enough along to be voted upon. This year your Committee determined to make a heroic struggle. After arranging for a little financial support, the fight began under the very able guidance of Senator J. J. McMaken, of Hamilton, O., and here let me say, this Society owes to this gentleman a debt of gratitude not easily paid. I hope you will see fit to remind him, in some manner, that you recognize the service he rendered you without the slightest reward to himself. As I said before, the fight began, and we had it hot and heavy for about four months—in the Senate. Early in April the bill passed the Senate with but one dissenting vote. The committee recognized that the worst of the fight was yet to come, as the House had declared itself as opposed to any legislation of the kind, and had emphasized this declaration by totally wrecking the medical bill, only a few days before our bill went over to them. We were repeatedly told by members of the house that it could not get through, but it did. Just about the time our bill got before the Committee from the House, Dr. Emminger was driven to Hot Springs by a severe illness, and soon afterward Dr. Taft was compelled to be at Ann Arbor, and your humble servant was left alone to meet the unterrified hosts of the House of Representatives, and I assure you that he felt about as lonely as ever before. After a deliberate survey of the situation, I decided to appeal to Dr. Grant Mollyneaux for help. He consented to do whatever was asked of him, and ask no questions. He kept his word, and I wish, in justice to him, to say that it was largely through influence he was able to bring to bear that we won the fight in the third round, and our bill became a law. I wish, officially, not as a member of the Committee on Dental law, but as President of the Ohio State Dental Society, to thank Dr. Mollyneaux for his most timely help. I do this because I am the only person who knows all that was done at that time.

There are many points about this law that I should like to discuss, but I pass them, expecting the report of the Committee to bring them out.

Ohio can now boast of five Dental colleges, and from this time forward a dentist who is only plain "Dr." will have to conduct himself in a very modest manner, speak in Dental meetings in a subdued voice, for there is liable to be present anywhere from twenty to thirty eagle-eyed professors ready to pounce upon every halting sentence he may utter, and totally annihilate everything that is not truly scientific.

The plain "Dr." will offer suggestions, not opinions, and ever keep in mind that he is surrounded by an atmosphere of great learning and intense dignity.

But there remains to us, of the common herd, one great privilege. We can, and most likely will, seek opportunity to tell the learned professors how they should conduct their colleges. We can tell them of the numerous acquirements the matriculate should possess. We can tell them how severe the preliminary examination should be. We can tell them how many years the student should be in college. We can tell them just what lines of study they should follow. We can tell them just how to conduct a clinic. We have never run a college, but we know all about it just the same. We can tell them how profoundly they should lecture the student on professional ethics. We can show them college and faculty advertisements that would make a circus advertising agent green with envy, and in this our great extremity we cry: "Oh, Allah—God *is* God, and Mohammed *is* his prophet, but Man is mighty uncertain!"

I desire to call your attention to an official act which the following correspondence will explain:

FOSTORIA, O., August 16, 1892.

*Dr. J. R. Callahan, Cincinnati, O.*

DEAR SIR:—I desire to call your attention to the fact that the Baltimore College of Dental Surgery gives the State Dental Society of each State the privilege of appointing one person as beneficiary student. See enclosed slip:

BENEFICIARY STUDENTS.—Each State Dental Society is privileged to send one Beneficiary student to this College at one-half the regular fees. This has been for some years an established feature of this College.

PROF. R. B. WINDER, DEAN,
*The Baltimore College of Dental Surgery,*
*No.* 716 *Park Avenue, Baltimore, Md.*

May I ask you to do what you can in my behalf, in regard to getting the appointment for me? Etc.

Yours Respectfully,

——— ———

To which I replied as follows:

——— ———

DEAR SIR:—In reply to your favor, in which you make application for the appointment by the State Society to what is known as the Beneficiary Student to the Baltimore Dental College, I have to say that we have five Dental colleges in Ohio, and I believe it to be the duty of the State Society, to support, in every legitimate way, the institutions within its own jurisdiction and not fall into the advertising schemes of colleges in other States. Yours Truly,

J. R. CALLAHAN,
*President Ohio State Dental Society.*

I have received several such letters in years gone by, but not being in a position to act I paid no attention to them. This year I report my action so that the society may reject or endorse my action, and thereby establish a precedent for those who may follow me in an official capacity.

And now, Brethren, the Executive Committee have prepared for us a feast of good things. It has required labor and patience to perfect the arrangements that have been made for us. We are indebted to Dr. Emminger and his committee for what is to come, and as we go along let us not forget to whom we are indebted for all these things.

"Behold how good and how pleasant it is for brethren to dwell together in unity! It is like the precious ointment upon the head—that ran down upon the beard, even Aaron's beard; that went down to the skirts of his garments, etc." If you find a member of this Society going about with a chip on his shoulder, seeking to have it knocked off, please recite, and don't fail to impress upon his mind this beautiful song of David.

